# Preimmunization with *Listeria*-vectored cervical cancer vaccine candidate strains can establish specific T-cell immune memory and prevent tumorigenesis

**DOI:** 10.1186/s12885-024-12046-7

**Published:** 2024-03-04

**Authors:** Yunwen Zhang, Sijing Liu, Mengdie Chen, Qian Ou, Sicheng Tian, Jing Tang, Zhiqun He, Zhaobin Chen, Chuan Wang

**Affiliations:** 1 West China School of Public Health and West China Fourth Hospital, Sichuan University, Chengdu, China; 2 Shen Zhen Biomed Alliance Biotech Group Co., Ltd., Shenzhen, China

**Keywords:** Tumor vaccine, Cervical cancer, Tumor prevention, Memory T cells, Immune response

## Abstract

**Background:**

Although HPV prophylactic vaccines can provide effective immune protection against high-risk HPV infection, studies have shown that the protective effect provided by them would decrease with the increased age of vaccination, and they are not recommended for those who are not in the appropriate age range for vaccination. Therefore, in those people who are not suitable for HPV prophylactic vaccines, it is worth considering establishing memory T-cell immunity to provide long-term immune surveillance and generate a rapid response against lesional cells to prevent tumorigenesis.

**Methods:**

In this study, healthy mice were preimmunized with LM∆E6E7 and LI∆E6E7, the two *Listeria*-vectored cervical cancer vaccine candidate strains constructed previously by our laboratory, and then inoculated with tumor cells 40 d later.

**Results:**

The results showed that preimmunization with LM∆E6E7 and LI∆E6E7 could establish protective memory T-cell immunity against tumor antigens in mice, which effectively eliminate tumor cells. 60% of mice preimmunized with vaccines did not develop tumors, and for the remaining mice, tumor growth was significantly inhibited. We found that preimmunization with vaccines may exert antitumor effects by promoting the enrichment of T cells at tumor site to exert specific immune responses, as well as inhibiting intratumoral angiogenesis and cell proliferation.

**Conclusion:**

Altogether, this study suggests that preimmunization with LM∆E6E7 and LI∆E6E7 can establish memory T-cell immunity against tumor antigens in vivo, which provides a viable plan for preventing tumorigenesis and inhibiting tumor progression.

**Supplementary Information:**

The online version contains supplementary material available at 10.1186/s12885-024-12046-7.

## Introduction

Cervical cancer is an infection-related cancer that is caused by persistent human papillomavirus (HPV) infection [1]. High-risk HPV type 16 or 18 can inhibit the antiviral immune response by expressing oncoproteins to inhibit interferon (IFN) secretion by keratinocytes and reducing the expression level of toll-like receptors (TLR9) after infecting the basal layer of the cervical epithelium so that the virus can exist in vivo for a long time. During persistent infection, the virus expresses E6 and E7 proteins to induce host cells carcinogenesis, resulting in tumorigenesis [2–4]. In general, the time span from persistent HPV infection to the development of cervical cancer is long, averaging 15 years [5].

HPV prophylactic vaccines provide effective immune protection against high-risk HPV infection. The memory B cells induced by HPV prophylactic vaccines can persistently produce high-affinity neutralizing antibodies, thus directly preventing the virus from infecting host cells [6]. Gardasil 9, in particular, provides protection against approximately 90% of cervical cancer cases caused by HPV infection and significantly reduces the incidence of other HPV-related diseases [7]. Studies have shown that the protection provided by HPV prophylactic vaccines decreases with the increased age of vaccination [8], and HPV prophylactic vaccines are not recommended for those who are not in the appropriate age range for vaccination. Moreover, because neutralizing antibodies cannot enter cells, the vaccines cannot provide protection for people are already infected with HPV or have a therapeutic effect on existing HPV-related diseases. The risk of HPV infection is lifelong. Although HPV can be actively cleared by the immune system in most cases, it is still capable of causing latent infection by inducing a defective immune response. Therefore, current HPV prophylactic vaccines still have protective limitations. It is worth considering establishing memory T-cell immunity to provide long-term immune surveillance and generate a rapid response against lesional cells to prevent HPV infection-related diseases such as cervical cancer.

Tumor vaccines are a promising immunotherapy method. They can effectively deliver tumor-associated antigens (TAAs) to the immune system by vaccine vectors and induce specific T-cell responses, especially CD8^+^ T-cell mediated CTL responses against TAAs, to clear cancerous cells. Memory T cells (Tm cells) are formed by the differentiation of effector T cells during the initial immune response, and the intracellular gene expression profile in Tm cells is rearranged. Tm cells have a shorter delay time and faster rate of cell division and can express different effector molecules simultaneously [9, 10]. Tm cells are not only present in immune organs but also distributed in peripheral organs for rapid responses. In addition, Tm cells can maintain long-term immune memory without repeat antigen stimulation, and the stable presence of Tm cells provides long-term immune surveillance [11–13]. Clinical studies have found that the proportion of CD45RO^+^CD8^+^ Tm cells infiltrated in tumor tissue was an independent factor in the prognosis in patients with squamous non-small cell lung cancer [14]. In a study by Li T et al., 40 d after treatment with nanovaccine in tumor-bearing mice, the increase in the proportion of CD8^+^ Tcm cells in peripheral blood was detected, and the levels of tumor necrosis factor-α (TNF-α) and IFN-γ were significantly upregulated after reinoculation of tumor cells, suggesting that CD8^+^ Tcm cells might recognize the reinvading tumor cells and thus elicit an antitumor immune response [15]. These results suggest that tumor vaccines have the potential to induce the formation of TAAs-specific Tm cells which are involved in antitumor immunity. The application of cervical cancer tumor vaccines for tumor prevention has been reported [16–18], but these studies did not evaluate the immune response induced by preimmunization with tumor vaccines, especially the Tm cells responses or explore the specific antitumor mechanism of tumor vaccines. Therefore, there are still gaps in the research on the immune regulation mechanism of cervical cancer tumor vaccines in tumor prevention. We have successfully constructed two *Listeria*-vector cervical cancer vaccine candidate strains, LM∆E6E7 and LI∆E6E7, and confirmed that combined immunotherapy with these two vaccine candidate strains could effectively inhibit tumor progression [19]. Therefore, in this study we aimed to investigate the role of TAAs-specific Tm-cell responses induced by preimmunizing with LM∆E6E7 and LI∆E6E7 in the tumor prevention and the potential antitumor mechanism.

## Materials and methods

### Mice, strains and cells

Specific pathogen-free (SPF) female C57BL/6J mice aged 6–8 weeks (Beijing Vital River Laboratory Animal Technology Co., Ltd. China) were housed in the SPF class animal house at the West China School of Public Health, Sichuan University. The animal experimental protocol was approved by the Ethics Committee of the Fourth West China Hospital of Sichuan University/West China School of Public Health (No. Gwll2022073).

TC-1 cells, derived from lung epithelial cells of C57BL/6J mice that were cotransformed with HPV-16 *E6*, *E7* and *ras* genes, were purchased from Beijing Silver Amethyst Biotechnology Co., Ltd., China.

LM∆, derived from *Listeria monocytogenes* (LM) 10403s, with knockouts of virulence genes *actA* and *plcB*, was constructed and preserved in our laboratory [20].

LM∆E6E7, a cervical cancer vaccine candidate strain expressing HPV-16 type E6 and E7 fusion protein with LM∆ as a vaccine vector, was constructed and preserved in our laboratory [19].

LI∆, derived from *Listeria ivanovii* (LI∆) PAM55, with knockouts of virulence genes *actA* and *plcB*, was constructed and preserved in our laboratory [21].

LI∆E6E7, a cervical cancer vaccine candidate strain expressing the HPV-16 type E6 and E7 fusion protein with LI∆ as a vaccine vector, was constructed and preserved in our laboratory [19].

### Regimen for tumor prevention by preimmunizing with LM∆E6E7 and LI∆E6E7

The cervical cancer vaccine candidate strains LM∆E6E7 and LI∆E6E7 and vaccine vector strains LM∆ and LI∆ were cultured in BHI medium (LAND BRIDGE, China, 37 °C, 180 rpm/min). Each strain was collected at the logarithmic growth stage, and bacterial suspensions were diluted with sterile PBS (Solarbio, China) to the LD_50_ determined by our laboratory (Supplementary material: Table S1) for inoculation.

**Fig. 1:**
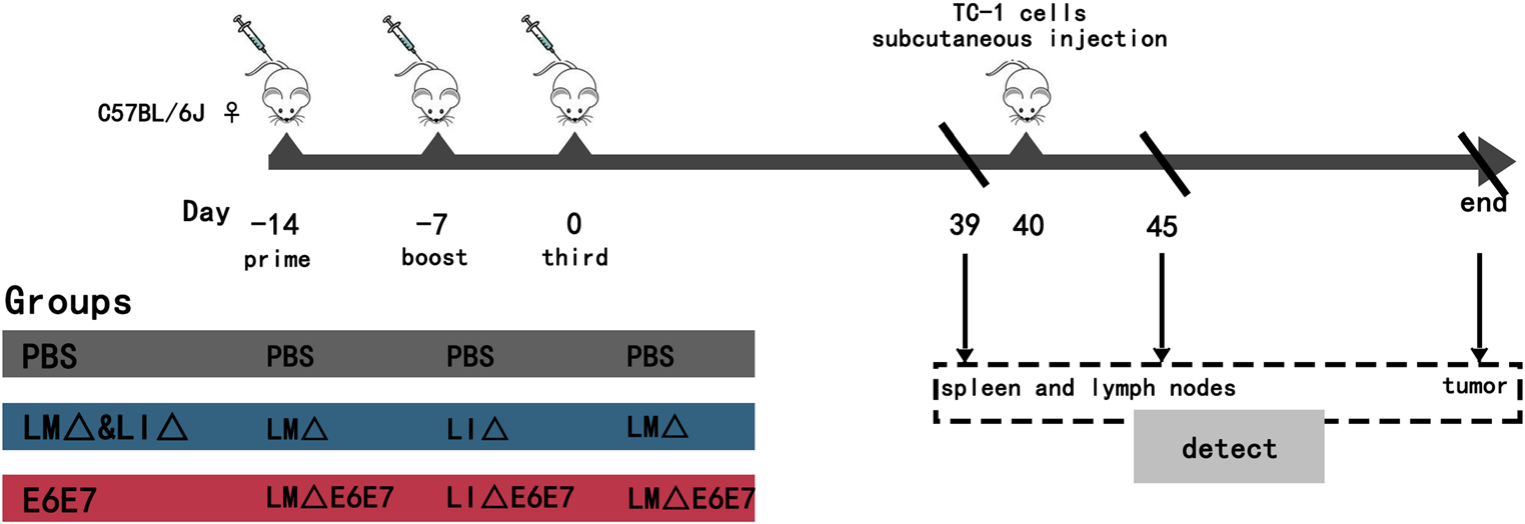
Animal experimental schedule

Mice were randomly divided into three groups: the PBS group (*n* = 22), vaccine vector strain group (LM∆ & LI∆, *n* = 22) and cervical cancer vaccine strain group (E6E7, *n* = 22). Mice in each group were injected intravenously (i.v.) with 100 µL of the corresponding strain suspensions or PBS. The regimen and experimental schedule are shown in Fig. 1. The day of completion of three immunizations was recorded as the 0th day, and all mice were housed normally. On the 40th day, each mouse was subcutaneously inoculated with 0.1 mL of 1 × 10^6^ cells/mL TC-1 cell suspension in the left abdomen. The longest and shortest diameters of the subcutaneous tumors were measured every two days using vernier calipers. When the longest diameter of the tumor reached 20 mm, it was defined as the observation endpoint of the tumor-bearing mice, and the mice were anesthetized by injecting intraperitoneally with 100 µL of 1% pentobarbital (Merck, USA) and then were sacrificed by spinal cord dislocation. On the 39th and 45th days, the mice were sacrificed by the same way, and the spleen and lymph nodes of mice in each group (*n* = 6) were collected to assess the proportions of immune cell subpopulations. At the observation endpoint, tumor tissues of the mice were collected to assess relevant indicators.

### Detection of immune cells in the spleen and lymph nodes by flow cytometry

Mouse spleen and lymph nodes were ground and filtered through 200-mesh sieves, and the cell suspension was obtained after adding erythrocyte lysate (an aqueous solution containing 0.0026 g/mL Tris and 0.0071 g/mL NH_4_Cl, pH = 7.2–7.4), incubating for 5 min at room temperature, and then washing twice with RPMI 1640 medium (HyClone, USA). The stimulating mixture was prepared as follows: the protein transport inhibitors (Thermo Fisher, USA) were diluted with 1640 medium containing 10% FBS (Gibco, USA) to twice the application concentration, and the peptides were added to a final concentration of 5 µg/mL. The peptides were synthesized by Sangon Biotech (Shanghai) Co., Ltd. The sequence information is shown in Supplementary material: Table S2. 100 µL of cell suspension was added to wells of a 96-well U-bottom plate with 100 µL of stimulation mix or a nonstimulating mixture (the mixture without peptides). The cells were incubated for 6 h in a cell incubator (Thermo Scientific, USA, 37 ℃, 5% CO_2_). Then, cell surface antibodies were added to the cell suspension and incubated at 4 °C for 30 min. After that, the cells were permeabilized with Cytofix/Cytoperm (BD PharMingen, USA) or Fixation/Permeabilization (eBioscience, USA), and anti-mouse CD32/16 antibody (eBioscience, USA) was added for blocking and incubated for 15 min at 4 °C. Intracellular or intranuclear antibodies were added and incubated at 4 °C for 45 min. After incubation, the cells were washed and resuspended in cell fixative buffer (BD PharMingen, USA) and assessed using flow cytometry with NovoCyte (Agilent, USA). Information on the antibodies is shown in Supplementary material: Table S3. The gating strategy for the flow cytometric analysis of immune cells (CD8^+^ T cells: CD3^+^CD8^+^, IFN-γ^+^CD8^+^ T cells: CD3^+^CD8^+^IFN-γ^+^, NK cells: CD11b^+^NK1.1^+^, MDSC: CD11b^+^Gr-1^+^, Treg: CD4^+^CD25^+^Foxp3^+^) in the spleen is shown in Supplementary material: Fig. S1. The gating strategy for the flow cytometric analysis of Tm cells (CD4^+^ T cells: CD3^+^CD4^+^, CD8^+^ T cells: CD3^+^CD8^+^, Tcm: CD44^+^CD62L^+^, Tem: CD44^+^CD62L^−^) in the spleen and lymph nodes is shown in Supplementary material: Fig. S2.

### RT‒qPCR

700 µL of RNA-easy (Vazyme, China) was added to tumor tissues (30 mg), and the tissues were ground (40 m/s, 20 s, twice) using a homogenizer (MP Biomedicals, USA). The homogenate was centrifuged at 13,000 rpm at 4 °C for 10 min, and the supernatant was pipetted into 2 mL EP tubes. The RNA was extracted according to the RNA-easy instructions, and then cDNA was obtained by reverse transcription according to the instructions of HiScript III All-in-one RT SuperMix Perfect for qPCR (Vazyme, China). qPCR was performed by using Ssofast EvaGreen Supermix (Biorad, USA), and the relative expression level of each gene was calculated according to 2^−∆∆Ct^. Information on the primers is shown in Supplementary material: Table S4 (Beijing Tsingke Biotech Co., Ltd.)

### Immunohistochemical staining

The exfoliated tumor tissues were immersed in tissue fixative (Solarbio, China) and fixed at room temperature for 24 h. Immunohistochemical staining (Ki67, CD3 and CD31) and histological section analysis of tumor tissues were performed by Sichuan Scientist Biotechnology Co., Ltd.

### Statistical analysis

Experimental data are shown as the mean ± standard deviation (mean ± SD). GraphPad 8.0 software was used for plotting, and SPSS 22.0 was used for statistical analysis. Tukey’s method was used when the data met the normal distribution and the variance was equal; otherwise, a nonparametric test was performed. *P* < 0.05 was considered statistically significant difference.

## Results

### Preimmunization with LM∆E6E7 and LI∆E6E7 prevented tumorigenesis and delayed tumor progression

**Fig. 2:**
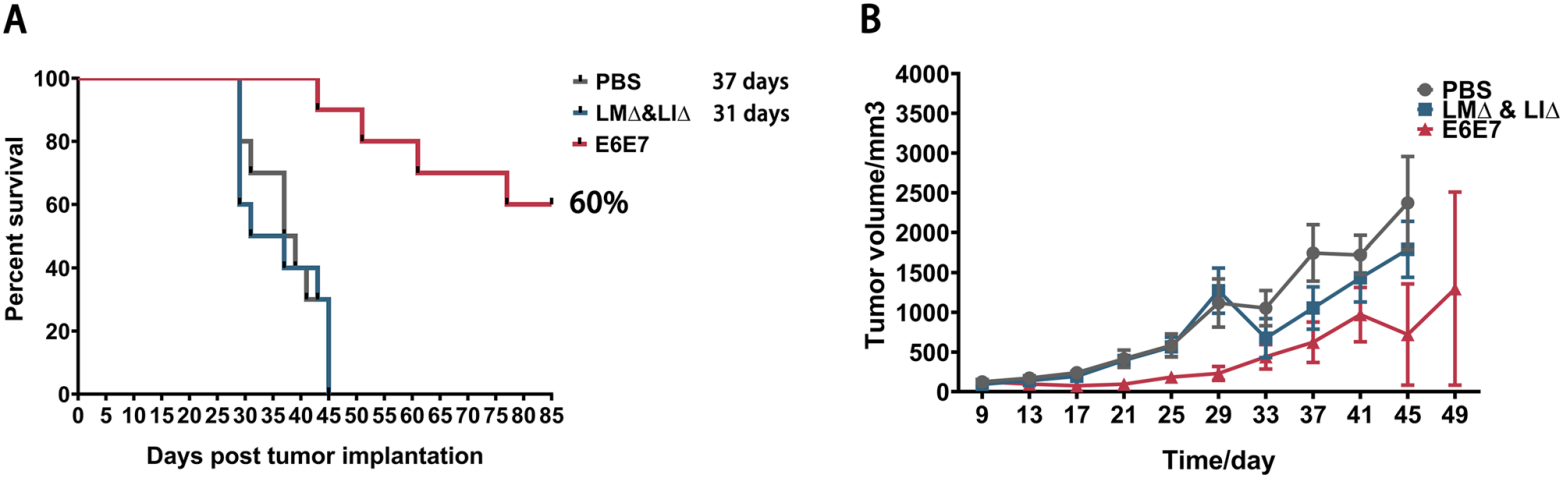
Preventive effect of preimmunization with LM∆E6E7 and LI∆E6E7 against tumorigenesis in mice. Survival time (**A**) and tumor volume (**B**) after subcutaneous inoculation of TC-1 cells in each group mice. PBS group (*n* = 10), LM∆ & LI∆ group (*n* = 10), E6E7 group (*n* = 10)

After inoculation of tumor cells, subcutaneous tumors were formed in the PBS and LM∆ & LI∆ groups mice, and the tumor volumes increased continuously until reaching the criteria for sacrifice. The median survival times for the PBS group and the LM∆ & LI∆ group were 37 d and 31 d respectively (Fig. 2A). For the E6E7 group (Fig. 2B), a rice-sized mass could be palpated in the abdomen approximately 7 d after inoculation of tumor cells. However, the masses in 60% of the E6E7 group mice gradually shrank to unpalpable levels within 2 weeks and remained tumor-free until the end of the observation period. The subcutaneous tumors of the remaining 40% of mice grew slowly until meeting the criteria for sacrifice, and the range of time of death was 43 d-77 d after tumor cells inoculation. Because the survival rate of mice in the E6E7 group was higher than 50%, the median survival time could not be calculated. The results showed that preimmunization with the cervical cancer vaccine candidate strains LM∆E6E7 and LI∆E6E7 had a preventive effect on tumorigenesis for healthy mice, with a 60% tumor-free protection rate, and significantly inhibited tumor progression.

### Preimmunization with LM∆E6E7 and LI∆E6E7 had no significant influence on the proportions of Tm cells subpopulations after stimulation by tumor cells

**Fig. 3:**
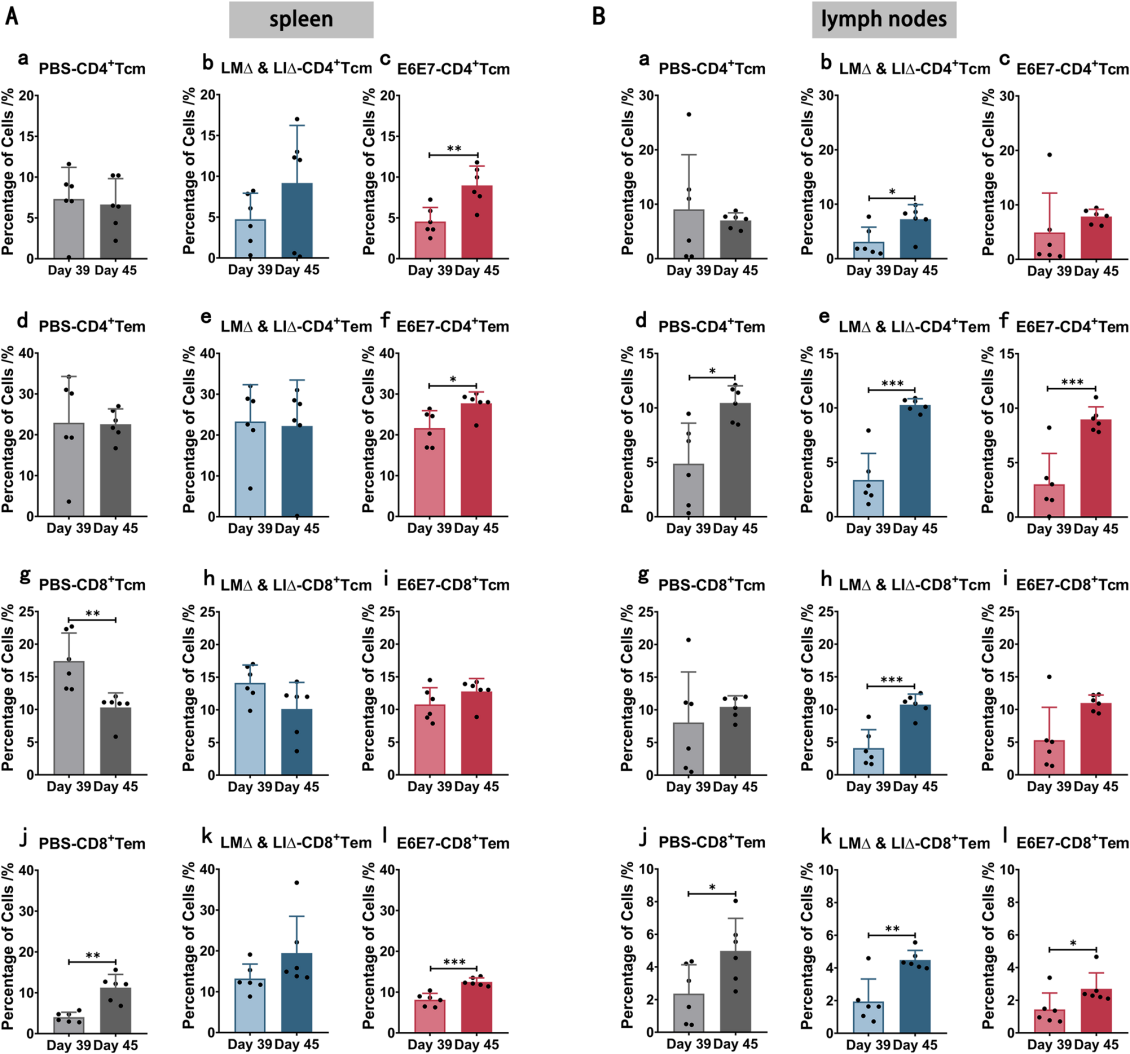
The proportions of Tm cells subpopulations in spleen and lymph nodes of mice before and after inoculation of TC-1 cells. The proportions of CD4^+^ Tcm, CD4^+^ Tem, CD8^+^ Tcm and CD8^+^ Tem cells in the spleen and lymph nodes of mice in each group were detected by flow cytometry before (Day 39) and after (Day 45) inoculation of TC-1 cells. (**A**): Comparison of the proportions of Tm cells subpopulations in the spleen of mice in each group before and after inoculation of TC-1 cells. (**B**): Comparison of the proportions of Tm cells subpopulations in the lymph nodes of mice in each group before and after inoculation of TC-1 cells. PBS group (*n* = 6), LM∆ & LI∆ group (*n* = 6), E6E7 group (*n* = 6), * *P* < 0.05, ** *P* < 0.01, *** *P* < 0.001

To evaluate whether preimmunization with cervical cancer vaccines had an effect on the cell proliferation of Tm cells subpopulations in vivo after antigen stimulation, we determined the proportions of Tm cells subpopulations in the spleen and lymph nodes of each group of mice before and after tumor cells inoculation (Fig. 3). In the spleen, compared with before the inoculation of TC-1 cells (Day 39), the proportions of CD4^+^ Tcm (*P* < 0.01), CD4^+^ Tem (*P* < 0.05) and CD8^+^ Tem (*P* < 0.001) cells were significantly higher in the E6E7 group of mice after inoculation (Day 45) (Fig. 3Ac, f, l); the proportion of CD8^+^ Tem cells (*P* < 0.01) in the PBS group was also significantly higher (Fig. 3Aj). In the lymph nodes, compared with before the inoculation (Day 39), mice in the E6E7 group had significantly higher proportions of CD4^+^ Tem (*P* < 0.001) and CD8^+^ Tem (*P* < 0.05) cells after the inoculation (Day 45) (Fig. 3Bf, l); the proportions of CD4^+^ Tem and CD8^+^ Tem cells were significantly increased in the PBS group (*P* < 0.05) (Fig. 3Bd, j), and the proportions of CD4^+^ Tcm, CD4^+^ Tem, CD8^+^ Tcm and CD8^+^ Tem cells were significantly higher (*P* < 0.05) in the LM∆ & LI∆ group (Fig. 3Bb, e, h, k). The results indicated that preimmunization with LM∆E6E7 and LI∆E6E7 did not significantly affect the proliferation levels of Tm cells subpopulations in the spleen and lymph nodes compared to the PBS and LM∆ & LI∆ groups.

### Antigen stimulation induced a specific CTL response in mice preimmunized with LM∆E6E7 and LI∆E6E7

**Fig. 4:**
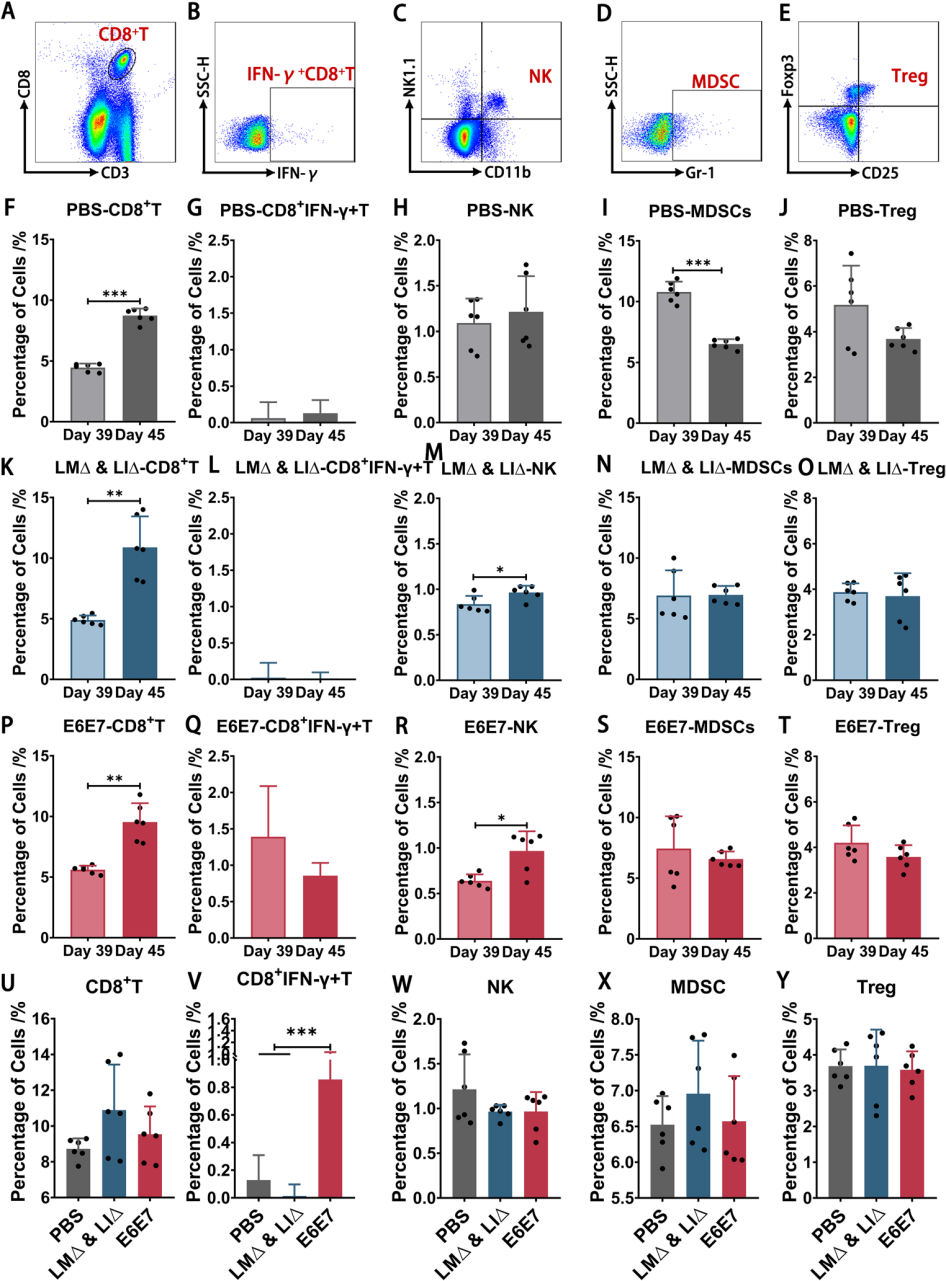
The proportions of immune cells in spleen of mice before and after inoculation of TC-1 cells. The representative flow scatter plots of immune cells (**A-E**). The proportions of CD8^+^ T cells, IFN-γ^+^CD8^+^ T cells, NK cells, MDSCs and Treg in the spleen of the PBS group (**F-J**), LM∆ & LI∆ group (**K-O**) and E6E7 group (**P-T**) before (Day 39) and after (Day 45) inoculation of TC-1 cells. Comparison of the proportions of immune cells in three groups in Day 45 (**U-Y**). PBS group (*n* = 6), LM∆ & LI∆ group (*n* = 6), E6E7 group (*n* = 6), * *P* < 0.05, ** *P* < 0.01, *** *P* < 0.001

Tumor cells inoculation induced immune responses in mice in vivo (Fig. 4). After TC-1 cells inoculation (Day 45), the proportions of CD8^+^ T cells in the spleen in the PBS group (*P* < 0.001), LM∆ & LI∆ group (*P* < 0.01) and E6E7 group (*P* < 0.01) were all significantly increased (Fig. 4A, F, K, P), and the proportions of NK cells in the E6E7 group and LM∆ & LI∆ group were also significantly increased (*P* < 0.05) (Fig. 4C, M, R). After the splenocytes were stimulated by tumor antigen epitopes, the CTL response was detected only in the E6E7 group (Fig. 4B, G, L, Q, V). And there was no difference in proportions of CD8^+^ T cells, NK, MDSC as well as Treg in three groups in Day 45 (Fig. 4A, C-E, U, W-Y). These results indicated that at the early stage of tumor formation, the proportions of NK cells and CD8^+^ T cells were increased in mice, similar to a spontaneous immune response. However, only the mice preimmunized with LM∆E6E7 and LI∆E6E7 were able to generate a specific CTL response against tumor antigens to block tumorigenesis.

### Preimmunization with LM∆E6E7 and LI∆E6E7 inhibited tumor growth by promoting the intratumoral antitumor immune response

**Fig. 5:**
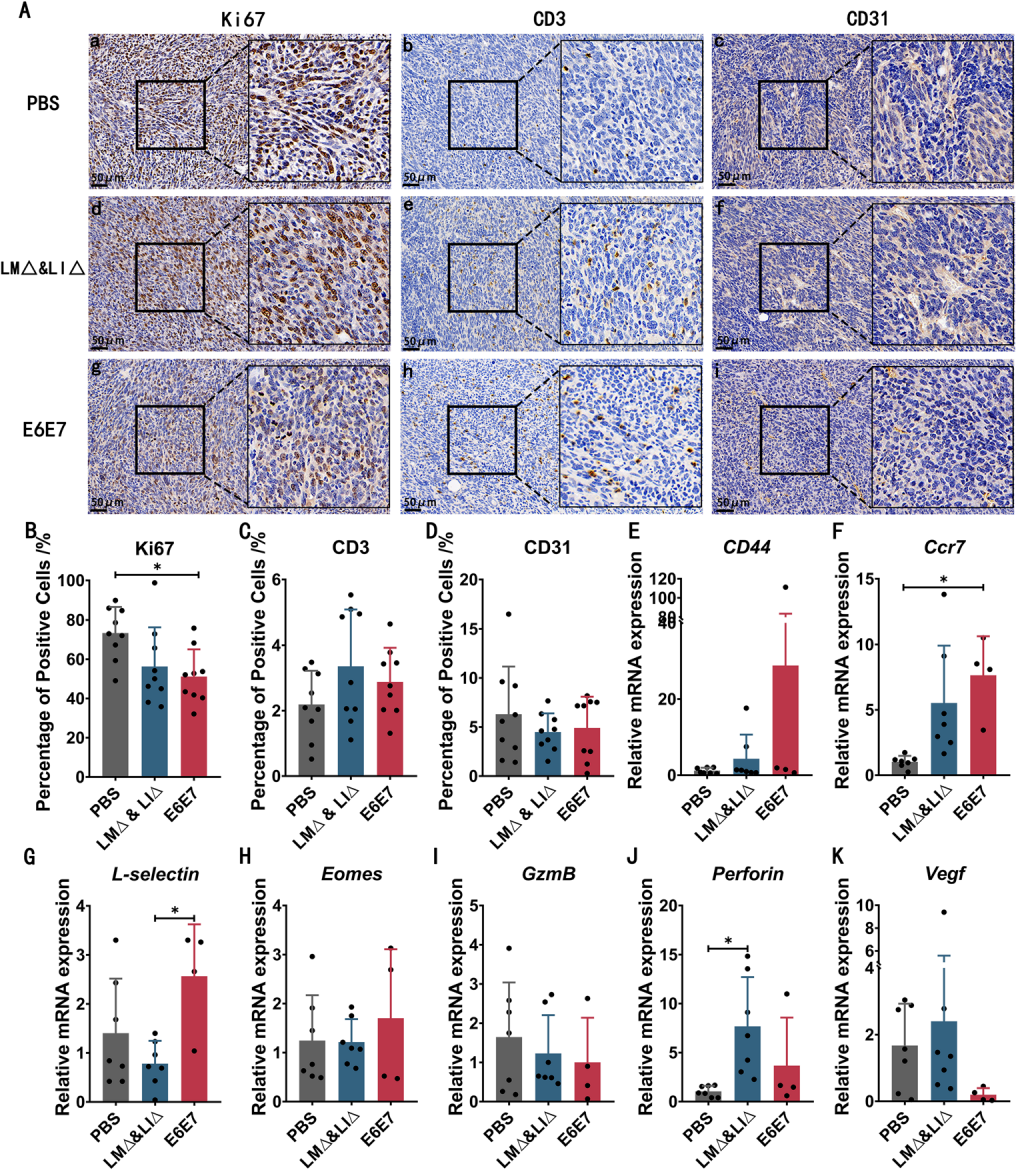
Analysis of the expression of immune molecule in tumor tissues. **A-D**: Immunohistochemical staining analysis of Ki67, CD3 and CD31 expression in mice tumor tissues. Tumor tissues were collected from each group and three fields of each section were randomly selected for analysis. PBS (*n* = 9), LM∆ & LI∆ (*n* = 9), and E6E7 (*n* = 9). E-K: the mRNA expression levels of *CD44* (**E**), *Ccr7* (**F**), *L-selectin* (**G**), *Eomes* (**H**), *GzmB* (**I**), *Perforin* (**J**) and *Vegf* (**K**) in tumor tissues were assessed by RT-qPCR. PBS group (*n* = 7), LM∆ & LI∆ group (*n* = 7), E6E7 group (*n* = 4), * *P* < 0.05

40% of mice in the E6E7 group developed tumors, but their tumors progressed significantly more slowly than those in the PBS group and the LM∆ & LI∆ group. Tumor tissues of tumor-bearing mice in each group were collected for immunohistochemical staining analysis at the endpoint (Fig. 5). The results showed that compared with the PBS group, the proportion of Ki67-positive cells infiltrated in the tumor tissues of the E6E7 group mice was significantly reduced (*P* < 0.05) (Fig. 5Aa, d, g, B), and it was consistent with tumor progress. The proportion of T cells infiltrated in the tumor tissue of the E6E7 and LM∆ & LI∆ group mice showed an increasing trend (*P* > 0.05) (Fig. 5Ab, e, h, C). While the proportion of CD31-positive cells showed a decreasing trend (*P* > 0.05) (Fig. 5Ac, f, i, D). The immune molecule expression levels in tumor tissues showed that the mRNA expression levels of *CD44* (*P* > 0.05), *Ccr7* (*P* < 0.05) and *L-selelctin* (*P* < 0.05) were increased in the E6E7 group compared with the PBS group or LM∆ & LI∆ group (Fig. 5E-G), and the mRNA expression level of *Eomes* was slightly higher (*P* > 0.05) (Fig. 5H). In addition, compared with the PBS group, the mRNA expression level of *Perforin* was slightly higher (*P* > 0.05) in the E6E7 group and significantly higher (*P* < 0.05) in the LM∆ & LI∆ group; compared with the PBS and LM ∆ & LI∆ groups, the expression level of *Vegf* was relatively reduced in the E6E7 group (*P* > 0.05) (Fig. 5I-K). The above results suggested that *Listeria* itself had a certain adjuvant effect and could enhance T-cell infiltration and reduce angiogenesis in tumor tissues. However, this adjuvant effect had no effect on tumor prevention. Only mice preimmunized with LM∆E6E7 and LI∆E6E7 could induce a tumor antigen-specific immune response to exert an effective antitumor effect.

## Discussion

T cells infiltrated in the tumor microenvironment (TME) cannot respond to antigen stimulation and kill tumor cells, but Tm cells isolated from the TME can be activated by tumor antigens and restart antitumor capability [22]. An increasing number of studies have shown a correlation between the Tm cells response and tumor disease progression. In tumor infiltrating lymphocytes (TILs) of patients with esophageal squamous cell carcinoma, the level of CD45RO^+^ Tm cells correlated with prognosis and tumor metastasis [23]. Enhancing the proportion of Tm cells at tumor sites in patients with glioblastomas might promote the therapeutic efficacy of dendritic cells vectored tumor vaccines [24]. Esquerre M et al. studied the protective effect of a therapeutic vaccine (GTL001) against HPV-16 and HPV-18 and found that tumor-bearing mice successfully cured by GTL001 produced a Tm-cell response against the reinoculated tumor cells to prevent tumor recurrence [25]. These findings suggest that Tm cells are directly involved in the antitumor immune responses and play important role in inhibiting tumor metastasis and maintaining long-term protective responses.

As the TAAs of HPV infection-associated cancers, E6 and E7 proteins are ideal target antigens for cervical cancer vaccines. In this study, the tumor antigens carried by LM∆E6E7 and LI∆E6E7 are formed by the cross-fusion of the E6 and E7 proteins of the HPV-16 type. Our study has shown that both strains have good immunogenicity and safety in mice [19]. In addition, we confirmed that the cross-immunization strategy, that is LM∆E6E7 as the first dose, LI∆E6E7 as the second dose and LM∆E6E7 as the third dose, could avoid the anti-carrier effect to obtain better tumor treatment effect than the single-strain immunization strategy (multiple immunizations with the same vaccine) [26]. In this study, healthy mice were cross-preimmunized with LM∆E6E7 and LI∆E6E7, and then we observed whether mice could resist the tumorigenesis induced by TC-1 cells. In reported studies on the tumor preventive effect of cervical cancer tumor vaccines [16–18], the time intervals between the completion of immunization and tumor cells inoculation in mice were varied, but none exceeded half a month. Study has found that after 40 days of immunization, CD8^+^ Tm cells in mice possess more stable and more definite immune function than those from earlier timepoints [27]. Due to the time interval is too short to form stable Tm-cell responses in mice, the observed results cannot fully represent the protective immune responses. Therefore, according to the time schedule rule of establishing immune memory in the body and to let the mice establish a stable immune memory response in vivo, we inoculated tumor cells at 40 d after completion of vaccine immunization, which can better simulate the process of vaccination against tumorigenesis in humans. This is the biggest difference between our study and the existing reports in terms of the study protocol of the tumor prevention effect of cervical cancer vaccines.

The mice in the PBS and LM∆ & LI∆ groups died successively due to tumor burden at one month approximately after tumor cells inoculation. However, 60% of mice that were preimmunized with LM∆E6E7 and LI∆E6E7 had no tumor burden until the endpoint (Fig. 2A). This indicates that direct immunization with cervical cancer tumor vaccines is able to induce protective immunity against tumors. After inoculation with tumor cells, the proportions of CD4^+^ Tcm, CD4^+^ Tem, CD8^+^ Tcm, and CD8^+^ Tem cells in the spleen and lymph nodes in all groups mice increased with different degrees (Fig. 3), and the proportions of CD8^+^ T cells and NK cells in the spleen were also increased. However, the proportion of IFN-γ^+^CD8^+^ T cells was significantly increased only in the E6E7 group after splenocytes were stimulated by tumor antigen epitopes (Fig. 4G, L, Q). IFN-γ is the basis of adaptive immunity, which plays an important antitumor role in the body. A study showed that tumor-bearing mice treated with tumor vaccines could clear the reimplanted tumor cells, and the Tem cells response has also been detected in vivo [28]. It suggested that only the mice that preimmunized with LM∆E6E7 and LI∆E6E7 were able to establish tumor antigen-specific T-cell immune memory which played an important role in inhibiting the tumor formation at early stage. But we lack further detection of immune response molecules in TME, such as perforin and granzyme B, which limiting the elaboration of tumor prevention mechanisms. Follow-up studies will focus on the immune response at this stage.

In general, tumor development induces the generation of high levels of regulatory T cells (Treg) and myeloid-derived suppressor cells (MDSCs) and thus forms a suppressive immune response that is favorable for tumor development [29, 30]. However, we did not find a significant increase in the proportions of Treg and MDSCs in the spleen of mice at 5 d after inoculation with tumor cells (Fig. 4X, Y). We think the reason may be that the detection timepoint (5 d post-inoculation in tumor cells) is too early to induce immunosuppression in vivo. In our other research, we continuously monitored the immune status of cervical cancer tumor-bearing mice and found that the elevation of the suppressive immune level occurred one week after tumor cells inoculation (data not published). Other studies have also reported that there is no difference in the proportions of Treg and MDSCs in the spleen at one week after tumor cells inoculation, but a significant increase could be detected two weeks later [31, 32]. Our previous study found that combined immunotherapy with LM∆E6E7 and LI∆E6E7 could significantly reduce the proportion of Treg at the tumor site [19], suggesting that preimmunization with LM∆E6E7 and LI∆E6E7 may also prevent tumor formation and delay tumor progression by weakening tumor-induced immunosuppression. In fact, the E6E7 group mice were not tumor-free for the entire process. Rice-sized masses formed after subcutaneous inoculation of TC-1 cells, but subcutaneous masses were completely cleared within two weeks in 60% of the mice. We speculate that at the early stage of tumor formation, an antitumor immune response was generated but was not sufficient to completely clear the tumor cells. With the persistence of the antitumor immune response and the weakness of the suppressive immune response later, the tumor cells were gradually cleared, and the mice remained tumor-free until the endpoint. In subsequent studies, the influence on the immune response caused by vaccines should be more fully explored by increasing the monitoring timepoints. It reminds us that there is no significant suppressive immune response in healthy women compared to cervical cancer patients. Tumor vaccines can induce a higher level of T cell response in healthy bodies than in cervical cancer patients, to generate stronger resistance against tumor antigens. This is also one of the key factors for women preimmunize with tumor vaccines in advance so as to prevent cancer.

Tm cells respond rapidly after stimulation by antigens. Tcm cells proliferate and differentiate into Tem cells. Tem cells exert immune effects and can localize to tumor sites to mediate protective responses. Immunohistochemical staining analysis showed that the cell proliferation rate in the tumor tissues of mice in the E6E7 group was significantly lower than that in the PBS group (Fig. 5A, B). CD44, L-selectin and CCR7 are expressed on the cell surface as signaling molecules that can drive the recruitment of T cells to inflammatory sites, activate leukocytes and promote the immune response [33–36]. In the TME, higher mRNA expression levels of *CD44*, *L-selectin* and *Ccr7* were detected in the E6E7 group mice than in the PBS group or the LM∆ & LI∆ group, and the proportion of infiltrated T cells was increased in the LM∆ & LI∆ and E6E7 groups (Fig. 5A, C, E-G). Angiogenesis in tumors is correlated with tumor growth and metastasis. Immunohistochemical analysis revealed a slight decrease in angiogenic capacity in the tumor tissues of mice in the E6E7 group, as well as a decrease in the expression level of *Vegf* (Fig. 5A, D, K). Activation of the transcription factor EOMES contributes to enhancing the clonal diversity of the memory pool [36], and a slightly higher expression level of *Eomes* was detected in the tumor tissues of mice in the E6E7 group than in the other two groups (Fig. 5H). These results indicate that the protective immunity induced by the preimmunizing cervical cancer vaccines LM∆E6E7 and LI∆E6E7 may inhibit tumor progression by promoting the enrichment of T cells at tumor sites to exert a specific immune response and by inhibiting intratumoral angiogenesis and cell proliferation. The specific effector molecules and mode remain to be clarified.

Our study suggests that preimmunization with cervical cancer vaccines can provide protection against tumor in healthy individuals. The prevalence of HPV infection among women without cervical abnormalities is 11.7% globally [37]. The implementation of effective preventive measures for cervical cancer, such as HPV vaccination and HPV screening, is severely limited in less developed countries and regions due to many factors, such as economic level, cultural ideology, and policy diffusion [38, 39], which also delays the early detection and treatment of cervical cancer. Pay attention to cervical cancer intervention has great significance to improve women’s health benefits. It is worth mentioning that Yvonne Paterson et al. studied on the vaccine LM-LLO-E7 suggests that the sustained expression of E6 and E7 within HPV carriers may induce a low affinity anti-tumor immune response to antigens through a tolerance mechanism. This also means that the protective effect of cervical cancer vaccines may be influenced by the expression levels of viral antigens in HPV infected individuals [40, 41]. Therefore, the protective effect of cervical cancer vaccines in people who are infected with HPV is still to be investigated.

## Conclusion

We explore the possibility of cervical cancer vaccines to be applied in tumor prevention from the perspective of disease prevention. Our study found that tumor vaccines induced the establishment of tumor antigen-specific T-cell immune memory in vivo. The appearance of tumor cells in the body triggered the high level of secondary immune response of Tm cells to eliminate tumor cells. At the same time, the specific T cells enriched at tumor sites inhibited intratumor angiogenesis and cell proliferation to prevent and delay cervical cancer. This study provides direct data to support that cervical cancer vaccines can be used to prevent tumors by preimmunization in healthy body, which may broaden the clinical application of tumor vaccines.

### Electronic supplementary material

Below is the link to the electronic supplementary material.

Supplementary Material 1

## Data Availability

The datasets analyzed during the current study are available from the corresponding author on reasonable request.

## Abbreviations

HPV: human papillomavirus
IFN: interferon
TLR: toll-like receptors
TAAs: tumor-associated antigens
Tm cell: memory T-cell
TNF-α: factor-α
SPF: specific pathogen-free
LM: *Listeria monocytogenes*
LI: *Listeria ivanovii*
TME: tumor microenvironment
TILs: tumor infiltrating lymphocytes
PBMCs: peripheral blood mononuclear cells
Treg: regulatory T cells
MDSCs: myeloid-derived suppressor cells
