# Supplementary material for: Preimmunization with Listeria-vectored cervical cancer vaccine candidate strains can establish specific T-cell immune memory and prevent tumorigenesis
Source: BMC Cancer. 2024 Mar 4;24:288. doi: 10.1186/s12885-024-12046-7 (PMC10910769; doi:10.1186/s12885-024-12046-7)
Supplement: Supplementary file 1 — Supplementary Material 1 [file 12885_2024_12046_MOESM1_ESM.docx]

Preimmunization with *Listeria*-vectored cervical cancer vaccine candidate strains can establish specific T-cell immune memory and prevent tumorigenesis

Yunwen Zhang^1,2#^, Sijing Liu^1#^, Mengdie Chen^2#^, Qian Ou^1^, Sicheng Tian^1^, Jing Tang^1^, Zhiqun He^1^, Zhaobin Chen^2*^, Chuan Wang^1*^

1 West China School of Public Health and West China Fourth Hospital, Sichuan University, Chengdu, China.

2 Shen Zhen Biomed Alliance Biotech Group Co., Ltd., Shenzhen, China

*Correspondence

Chuan Wang: [wangchuan@scu.edu.cn](mailto:wangchuan@scu.edu.cn).

Zhaobin Chen: [chenzb.md@vip.163.com](mailto:chenzb.md@vip.163.com).

^#^ Yunwen Zhang, Sijing Liu, Mengdie Chen contributed equally to this work.

**Supplementary Table**

Table S1. The concentration and inoculum volume of each strain.

| Strain | LD_50_ | Concentration | Inoculum volume |
| --- | --- | --- | --- |
| LM∆ | 5×10^7^ cfu/mL | 5×10^7^ cfu/mL | 100 μL |
| LI∆ | 2×10^8^ cfu/mL | 2×10^8^ cfu/mL | 100 μL |
| LM∆E6E7 | 1.3×10^8^ cfu/mL | 1.3×10^8^ cfu/mL | 100 μL |
| LI∆E6E7 | 4×10^8^ cfu/mL | 4×10^8^ cfu/mL | 100 μL |

Table S2. The information of peptides.

| Peptide | Amino acid sequence |
| --- | --- |
| HPV16 E6E7-1 | RAHYNIVTF |
| HPV16 E6E7-4 | YDFAFRDL |

Table S3. The information of antibody used in flow cytometry.

| Name | Clone No | Company |
| --- | --- | --- |
| FITC Rat Anti-Mouse CD3 | 17A2 | BD PharMingen, USA |
| FITC Rat Anti-Mouse CD4 | RM4-5 | BD PharMingen, USA |
| PE Rat Anti-Mouse CD8a | 53-6.7 | BD PharMingen, USA |
| APC-CyTM7 Anti-Mouse CD44 | IM7 | BD PharMingen, USA |
| APC Anti-Mouse CD62L | MEL-14 | BD PharMingen, USA |
| APC Anti-mouse IFN-γ Antibody | XMG1.2 | eBioscience, USA |
| FITC Anti-mouse CD11b Antibody | M1/70 | eBioscience, USA |
| PE Anti-mouse NK1.1 Antibody | PK136 | eBioscience, USA |
| PerCP Cy5.5 Anti-mouse Gr-1 Antibody | R86-BC5 | eBioscience, USA |
| PE Anti-mouse FOXP3 Antibody | FJK-16s | eBioscience, USA |
| APC Anti-mouse CD25 Antibody | PC61.5 | eBioscience, USA |
| Anti-mouse CD32/16 Purified | 93 | eBioscience, USA |

Table S4. Primer sequence information of genes.

| Gene | Forward primers | Reverse primers |
| --- | --- | --- |
| *Gapdh* | ACCCAGAAGACTGTGGATGG | ACACATTGGGGGTAGGAACA |
| *CD44* | CAGCCTACTGCAGCTCCAAA | CGCCGCTCTTAGTGCTAGAT |
| *L-selectin* | AGCCAATCTGCCAAGAGACA | CGAGAATGCGGTGACCATGA |
| *Ccr7* | GCGGAACAAGGCCATCAAGG | GACGGAGGCCAGGCTGTAGG |
| *Eomes* | GGACAATAACATGCAGGGCAA | TGTGCAGAGACTGCAACACT |
| *Perforin* | CAGGTCAACATAGGCATCCACG | GAACAGCAGGTCGTTAATGGAG |
| *GzmB* | AGCAGCCTGAGGCGATAT | CTGGGTCTTCTCCTGTTCTT |
| *Vegf* | CGAACGTACTTGCAGATGTGAC | GCGTGGTGGTGACATGGTTA |

**Supplementary Figure**


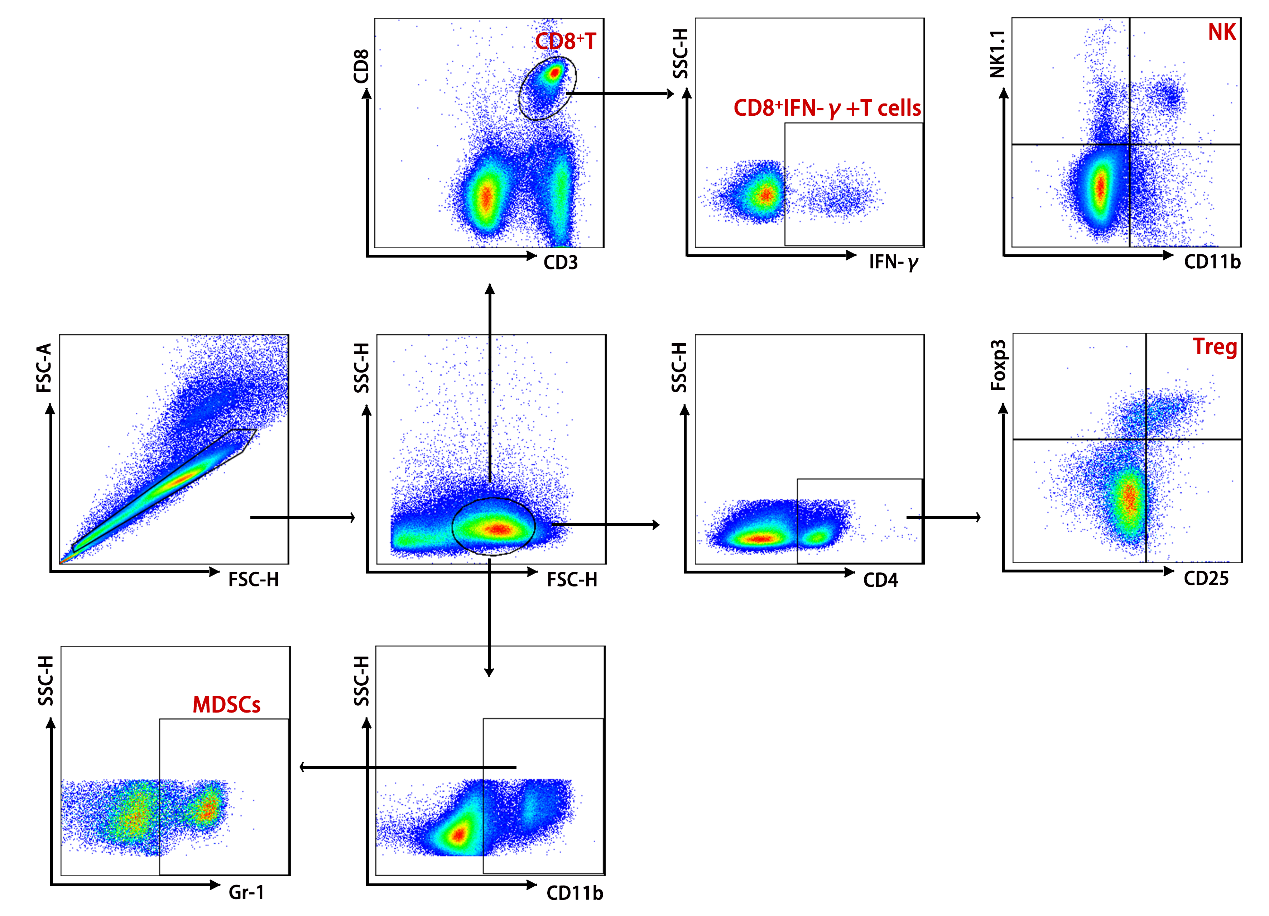


**Fig.** S1 Gating strategy of flow cytometric analysis of immune cells in the spleen of mice.


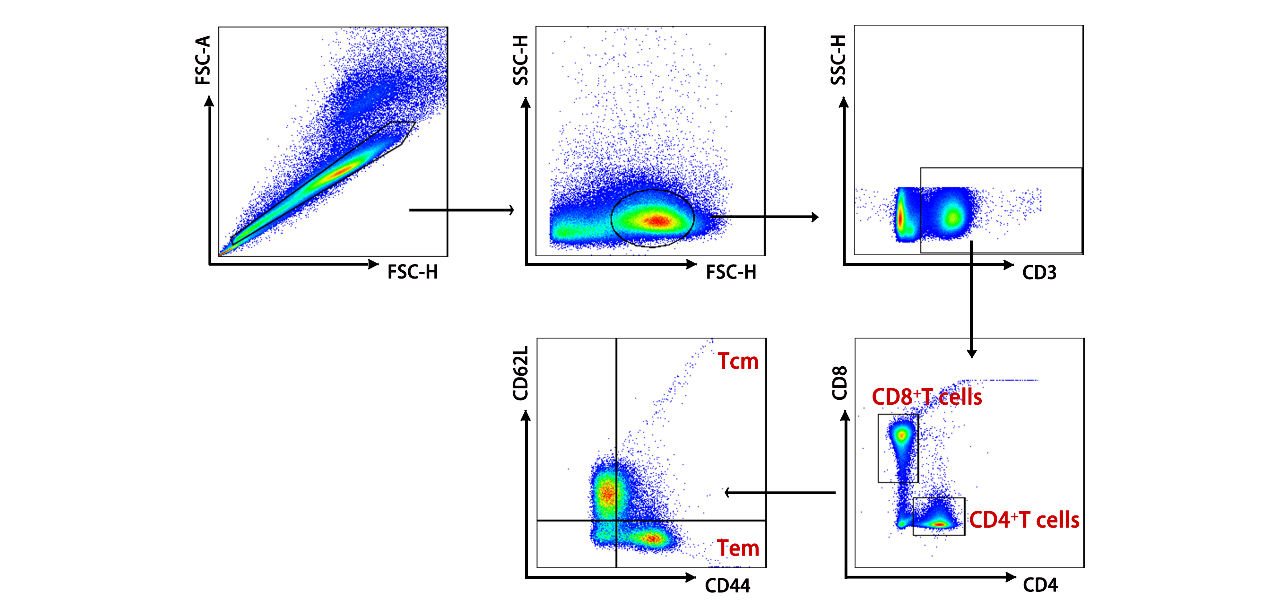


**Fig. S2** Gating strategy of flow cytometric analysis of Tm cells in the spleen and lymph nodes of mice.
